# Radiologic diagnostic procedures in severely injured patients - is only whole-body multislice computed tomography the answer?

**DOI:** 10.1186/s12245-015-0053-8

**Published:** 2015-02-28

**Authors:** Tobias Topp, Rolf Lefering, Caroline L Lopez, Steffen Ruchholtz, Wolfgang Ertel, Christian A Kühne

**Affiliations:** Department of Orthopedic, Trauma, Hand and Reconstructive Surgery, Charité Berlin, Campus Benjamin Franklin, Hindenburgdamm 30, 12203 Berlin, Germany; Institute for research in operative medicine (IFOM), University of Witten/Herdecke, Ostmeerheimer Str. 200, Haus 38, 51109 Cologne, Germany; Department of General Surgery, University Hospital Giessen and Marburg, Baldingerstraße, 35043 Marburg, Germany; Department of Trauma, Hand and Reconstructive Surgery, University Hospital Giessen and Marburg, Baldingerstraße, 35043 Marburg, Germany

**Keywords:** Blunt trauma, Polytrauma, Multislice CT, Whole body CT, Radiological diagnostics

## Abstract

**Background:**

Whole-body multislice computed tomography (WB-MSCT) has become an important diagnostic tool in the early treatment phase of severely injured patients. The optimal moment of WB-MSCT’s use during this treatment phase remains unclear. Many trauma centers use WB-MSCT in addition to conventional radiographs, while some trauma centers use WB-MSCT as the only radiological tool. The aim of this study was to determine the differences between these two protocols and to answer the question of whether conventional radiographs can still be used in the safe treatment of polytrauma patients.

**Methods:**

Patients from the TraumaRegister DGU® with an injury severity score (ISS) of ≥16 were included. Group I received conventional radiographs and focused assessment with sonography in trauma (FAST) prior to a WB-MSCT, and group II received an initial WB-MSCT and FAST. Both groups were compared concerning treatment time and outcome.

**Results:**

A total of 3,995 patients in group I were compared to 4,025 patients in group II. There were no differences in ISS (29.97 vs. 29.94), gender (male: 73.5% vs. 72.8%), age (45.47 vs. 45.12 years), or calculated mortality (21.41% vs. 21.44%). Time needed in the resuscitation room was slightly longer in group I (72 vs. 64 min); the durations until admittance to the ICU and arrival to the OR were not significantly different between the groups. There was no difference in mortality (18.2% vs. 18.4%) or the standardized mortality ratio (SMR) (0.85 vs. 0.86).

**Conclusions:**

WB-MSCT plays an inherent role in the treatment of multiple-injured patients. However, the use of WB-MSCT as the only diagnostic method in the resuscitation room is not needed. Conventional radiographs and FAST followed by WB-MSCT can be performed in the early resuscitation phase without impairing patient outcomes. This approach enables the emergency room team to perform life-saving procedures - chest-tube insertion, laparotomy, cardiopulmonary resuscitation -immediately and simultaneous. Nevertheless, randomized multi-center trials are needed to determine the comparability and effectiveness of these algorithms.

## Background

The initial management of severely injured patients in the resuscitation room can be challenging for the trauma team. There are approximately 40,000 severely injured patients resulting from motor vehicle accidents, high falls, violence, or suicide attempts in Germany per year. To guarantee immediate rescue and prompt treatment of these patients, there are approximately 700 trauma centers of different designations - i.e., supraregional, regional, and local trauma centers.

Multislice computed tomography (MSCT) of the head, neck, chest, abdomen, and pelvis (whole-body MSCT) is an established diagnostic tool in the modern treatment of severely injured patients during the resuscitation room phase [1-3].

According to Huber-Wagner et al., there is evidence that the immediate use of whole-body multislice CT (WB-MSCT) in trauma care significantly increases the probability of survival in patients with severe trauma [1]. Some studies that compared the first line use of WB-MSCT to a standard protocol using conventional radiographs prior to organ-focused CT found shorter times for diagnostic workup completion when a WB-MSCT was used as the first and only diagnostic tool [3].

To date there have been no randomized, prospective studies on this topic. The REACT-2 trial attempted to ‘provide evidence on the value of immediate total-body CT scanning during the primary survey of severely injured trauma patients’ [4], but MSCT was compared to conventional radiographs with additional conventional CT and not to conventional radiographs performed prior to MSCT. Most trauma centers follow a protocol that uses an initial WB-MSCT or conventional radiographs followed by an organ-focused CT for the diagnosis in severely injured patients [5]. There is no doubt about the advantages of WB-MSCT scans in detecting head injuries or injuries to solid and hollow *viscera* of the chest or the abdomen. Due to this fact, conventional radiographs cannot replace CT scans in the diagnostic algorithm of severely injured patients but are useful to detect the site of life-threatening injuries more quickly. In addition, there is no need to interrupt other required interventions during radiologic diagnosis - i.e., chest tube insertion, intubation, or mechanical cardiopulmonary resuscitation. Furthermore, the trauma team does not have to leave the resuscitation room for conventional radiographs, but they do need to leave for WB-MSCT. Lögters et al. [6] analyzed the data of 12,971 patients from TraumaRegister DGU® of the German Trauma Society regarding the incidence and causes of life-threatening injuries in major trauma patients (ISS ≥16). In 5.5% (*n* = 713) of the patients, the diagnostic algorithm in the resuscitation room was interrupted because of emergency surgery. In this patient group, the incidence of severe abdominal injuries was fourfold higher than that among the remaining patients, while severe pelvic injuries had a twofold higher incidence [6].

In addition to the fact that there are differences in the structural, personnel, and apparative requirements for different levels of treatment, all of these trauma centers have well-educated trauma teams and standardized equipped resuscitation rooms for the early treatment phase of trauma patients. For example, either X-ray or computed tomography scanners are located in every resuscitation room.

Since CT was introduced into the initial diagnostic workup, it has gained increasing importance for the early treatment phase of trauma care. With the development of MSCT and the increasing application of WB-MSCT, diagnostic accuracy has increased. Computed tomography is more precise for detecting even small injuries and aids in establishing plans for further therapy. Due to this fact, more and more authors believe that computed tomography is the diagnostic tool of choice for severely injured patients and is superior to other examination protocols [7-10]. The possible advantages of WB-MSCT are the fast imaging of the body regions of primary interest (abdomen, thorax, brain, spine, and pelvis) and the early definition of a treatment plan. Possible disadvantages are that WB-MSCT results in higher radiation doses - especially in patients with only minor injuries but with suspected severe injuries - and impairs the simultaneous performance of lifesaving procedures - i.e., chest tube or vessel catheter insertion, resuscitation with cardiac massage, or laparotomy/thoracotomy.

A recent survey of trauma centers in Germany revealed that only 21% of all supraregional and 5% of all regional trauma centers had a CT scanner located in the resuscitation room itself. For local trauma centers, this same figure was only 0.5%. The remaining trauma centers had a CT scanner either near the resuscitation room or on the next floor. These results show that WB-MSCT is not available as a first diagnostic tool in many hospitals. The majority of hospitals must use conventional radiographs prior to WB-MSCT, which leads to the question of possible advantages and disadvantages of these protocols. To date, no study has analyzed this question in patients who require a WB-MSCT because of their mechanism of injury. One of the most discussed questions is whether using WB-MSCT as the first diagnostic tool leads to decreased mortality.

The objective of this study was to compare a protocol that uses WB-MSCT as the first and only diagnostic tool to a protocol that uses conventional radiographs prior to WB-MSCT regarding a) duration of the initial treatment phase in the resuscitation room, b) length of stay in the intensive care unit (ICU), c) ventilation days, d) length of hospital stay, and e) mortality.

## Methods

The TraumaRegister DGU® of the German Trauma Society (TR-DGU) was founded in 1993. This registry prospectively collects anonymous data from severely injured patients. Up to 2009, data from 51,425 trauma patients from 266 hospitals were collected. The documentation covers four phases after trauma: A, pre-hospital phase; B, resuscitation room and initial therapy until ICU admission; C, ICU; and D, discharge including all injuries, relevant procedures, and patient outcomes. All trauma patients who reached the hospital alive were treated in the resuscitation room and required care in an ICU (including those who died before reaching the ICU) are included in the TR-DGU. Data are submitted to a central database server, and the anonymity of the patients and trauma center is guaranteed. Epidemiological, physiological, laboratory, diagnostic, operative, interventional, and intensive care medical data are recorded. Since 2002, data input has been based on an online documentation system (www.traumaregister.de).

All primary admitted patients with an ISS of ≥16 and with documented times of conventional radiographs and WB-MSCT diagnosis were included. Patients with penetrating injuries were excluded. Patients were divided into two groups. Group I received an initial WB-MSCT, mostly with the scanner located in the resuscitation room, and group II received a conventional radiograph prior to WB-MSCT. Both groups also received an initial FAST ultrasound. The two groups were compared concerning length of stay in the resuscitation room, time until admission to the OR or ICU, gender, age, Glasgow Coma Scale (GCS), mortality, required surgery, injury pattern, shock, mechanism of injury, length of stay in the ICU and the hospital, and the number of transfused packed red blood cells (PRBC). The prognoses of the patients were derived using the Revised Injury Severity Classification (RISC) score [11], and the standardized mortality ratio (SMR; ratio of recorded to expected mortality) was calculated. This score was developed with data from 2,009 patients in the TraumaRegister DGU® (1993 to 2000) and has been validated for 3,475 patients (2001 to 2003). RISC score-adjusted outcome comparisons have been routinely reported every year by the Trauma Registry since 2003 [11].

Data are presented as the mean ± standard deviation (SD) and were analyzed with the *t*-test for continuous variables. Categorical variables are presented as percentages and were analyzed with the chi-squared test. A *p* value <0.05 was considered significant. Data were analyzed with SPSS statistical software (Version 21.0, IBM Inc., Armonk, NY, USA).

## Results

From 1993 to 2009, 18,268 severely injured trauma patients with an ISS of ≥16 and age of ≥16 who required urgent resuscitation room treatment and radiological diagnostics were reviewed from the TraumaRegister DGU® database. Among these patients, 1,195 (6.5%) underwent conventional radiographs alone, 4,971 (27.2%) had a CT-scan without additional conventional radiographs, and 12,102 (66.2%) had computed tomography and conventional radiographs. In 8,517 of the 12,102 cases, the performance time was documented for the conventional radiographs and CT scans. A total of 91.1% (*n* = 7,764) of these patients had an initial conventional radiograph - mostly an X-ray of the thorax and the pelvis - followed by a CT-scan. In 3,995 patients (52%), this CT scan was performed as a whole body multislice CT (WB-MSCT) scan (group I). The remaining 3,736 patients underwent CT scans of particular body parts or CT scans were not performed as a WB-MSCT. In patients receiving the CT diagnosis alone, 4,025 (82%) had a WB-MSCT (group II) and 918 patients underwent a CT other than WB-MSCT. For further analysis, all patients with initial conventional radiographs followed by a WB-MSCT scan (group I) were compared with patients who underwent WB-MSCT as the initial diagnostic tool alone (group II).

Patients receiving an initial conventional radiograph prior to the MSCT-scan (group I) had the same ISS of 30.0 as group II (WB-MSCT-scan alone) (see Table 1). Differences in gender, age, surgical procedures, mortality, duration of hospitalization, stay in the ICU, ventilation-free days, multiorgan failure, and transfused packed red blood cells (PRBC) are illustrated in Table 1. Slightly more severe head and thoracic injuries (abbreviated injury scale (AIS) ≥3) were found in group II. Severe abdominal and extremity trauma was slightly lower in group II (see Table 2). The mortality within the first 24 h was almost equal in both groups (9.7%, *n* = 395 vs. 9.5%, *n* = 382). Patients in group II had significantly shorter treatment times in the resuscitation room than patients in group I, despite the fact that duration until admittance to the ICU or to the OR did not differ significantly between the groups (see Table 3). Additionally, the SMR did not differ significantly between the groups (0.85 vs. 0.86; *p* = 0.910).

**Table 1:** **Comparison between group I (initial X**-**ray followed by MSCT) and group II (initial MSCT)**

	**X**-**ray followed by WB**-**MSCT** **(group I)**	**Initial WB**-**MSCT without X**-**ray** **(group II)**
Male (*p* = 0.497) chi^2^ = 0.462	73.5% (*n* = 2,937)	72.8% (*n* = 2,932)
Age (*p* = 0.429)	45.5 (±20) years	45.1 (±19.8) years
ISS (*p* = 0.913)	29.9 (±12.3)	29.9 (±12.6)
Surgery (*p* = 0.550) chi^2^ = 0.357	74.4% (*n* = 2,998)	73.8% (*n* = 2,993)
Mortality (*p* = 0.786) chi^2^ = 0.074	18.2% (*n* = 732)	18.4% (*n* = 746)
Hospitalization (*p* < 0.001)	25.2 (±25.2) days	27.9 (±30.2) days
ICU (*p* < 0.01)	12.3 (±14.3) days	13.0 (±15.4) days
Ventilation days (*p* < 0.01)	7.7 (±11.7)	8.7 (±13.4)
Red packed blood cells (*p* = 0.498)	2.3 (±5.7)	2.4 (±6.2)
RISC (*p* = 0.961)	21.4 % (±28)	21.4 % (±28)

ISS = injury severity score; WB-MSCT = whole-body multislice computed tomography; ICU = intensive care unit; RISC = revised injury severity classification.

**Table 2:** **Comparison between patients in groups I and II concerning the injury pattern of severe injuries** (**AIS** ≥**3**)

	**X**-**ray followed by WB**-**MSCT** **(group I)**	**Initial WB**-**MSCT without X**-**ray** **(group II)**
AIS_head_ (*p* < 0.05)	55.1% *n* = 2,220	57.7 % *n* = 2,338
AIS_thorax_ (*p* = 0.501)	63.5% *n* = 2,558	64.2 % *n* = 2,603
AIS_abdomen_ (*p* < 0.05)	23.4% *n* = 944	21.6% *n* = 877
AIS_extremities_ (*p* < 0.01)	41.4% *n* = 1,667	38.1% *n* = 1,543

AIS = abbreviated injury scale; WB-MSCT = whole-body multislice computed tomography.

**Table 3:** **Comparison of patients in groups I and II concerning different durations needed during patients treatment**

	**X**-**ray followed by WB**-**MSCT** **(group I)** **(min)**	**Initial WB**-**MSCT without X**-**ray** **(group II)** **(min)**
Time in RR (*p* < 0.001)	72 (±40)	64 (±39)
Admittance on ICU (*p* = 1.0)	197 (±149)	197 (±147)
Arrival in the OR (*p* = 0.687)	144 (±187)	141(±203)

RR = resuscitation room; ICU = intensive care unit; OR = operation room; WB-MSCT = whole-body multislice computed tomography.

### Injury severity and mortality

As expected, the stratification of the injury severity (ISS 16 to 14, ISS 25 to 49, and ISS ≥50) revealed a clear correlation with mortality rate. Significant differences in mortality between groups I and II were not shown after stratification for ISS (see Table 4).

**Table 4:** **Injury patterns** (**AIS** ≥**3**) **in patients with different injury severity levels in groups I and II**

	**ISS**	**AIS >** **2**				
		**Head**	**Thorax**	**Abdomen**	**Extremities**	
X-ray followed by WB-MSCT (group I)	16 to 24	36.6%	49.9%	11.8%	34.2%	4.3%
	25 to 49	64.2%	69.5%	27.5%	43.5%	21.7%
	50+	83.2%	88.1%	50.9%	60.5%	58.8%
Initial WB-MSCT without X-ray (group II)	16 to 24	39.4 % (*p* = 0.105) chi^2^ = 2,624	51.9% (*p* = 0.253) chi^2^ = 1.308	11.0% (*p* = 0.465) chi^2^ = 0.534	31.8% (*p* = 0.149) chi^2^ = 2.085	4.5% (*p* = 0.728) chi^2^ = 0.121
	25 to 49	66.6% (*p* = 0.096) chi^2^ = 2.771	70.0% (*p* = 0.717) chi^2^ = 0.131	24.9% (*p* = 0.058) chi^2^ = 3.585	39.8% (*p* < 0.05) chi^2^ = 6.011	21.5% (*p* = 0.897) chi^2^ = 0.017
	50+	84.5% (*p* = 0.650) chi^2^ = 0.205	83.4% (*p* = 0.074) chi^2^ = 3.196	48.5% (*p* = 0.526) chi^2^ = 0. 402	55.1% (*p* = 0.145) chi^2^ = 2.120	60.1% (*p* = 0.723) chi^2^ = 0.126

AIS = abbreviated injury scale; ISS = injury severity score; WB-MSCT = whole-body multislice computed tomography.

## Discussion

### Outcome

The expected mortality rate after sustained severe trauma, based on the RISC score, was similar between group I and group II (18.2% vs. 18.4%, *p* = 0.8). Both groups were comparable regarding gender, age, and injury severity (ISS) (see Table 1). Patients with conventional radiography, FAST, and WB-MSCT (group I) had shorter ICU and hospital stays compared to patients with WB-MSCT only (group II) (see Table 1).

To determine if one of these concepts has advantages concerning the treatment of patients with different levels of injury severity, three subgroups were compared ( ISS 16 to 24. ISS 25 to 49, ISS ≥50). We did not find significant differences in mortality or injury patterns between patients with an initial conventional radiograph followed by WB-MSCT compared to an initial WB-MSCT for the three subgroups with an ISS 16 to 24, ISS 25 to 49, and ISS ≥50. These results show that even patients with an ISS ≥50 are not at a disadvantage because of an initial conventional radiograph radiography.

There is strong evidence that WB-MSCT yields in a lower mortality and better outcome than emergency room diagnostics with selective CT only in multiple injured patients. In a recent published meta-analysis by Jiang et al. [12], the data of 26,371 major trauma patients from 11 trials were analyzed. The authors found that for early diagnosis of major trauma patients the whole-body computed tomography was associated with a decreased mortality compared with selective CT strategies [12].

To our opinion, the obvious advantage of initial X-ray diagnosis of the chest and pelvis, simultaneous to the FAST is the possibility to perform life-saving procedures like chest-tube insertion, thoracotomy, cardiopulmonary resuscitation - immediately and simultaneous. After stabilization, definite diagnosis can be done by WB-MSCT. Therefore, the ability to perform X-ray imaging is essential in the resuscitation room - even in the presence of a CT scanner.

Especially in countries with higher amount of penetrating injuries like stab or gunshot, the need of immediate surgical interventions the initial diagnosis with only WB-MSCT can waste precious time, because life-saving procedures are not possible during the CT scanning phase.

### Radiation exposure and scanning time

The American College of Surgeons Committee on Trauma (ACS COT) stated that up to 40% of severely injured trauma patients are overtriaged [13]. Analysis of the TR-DGU showed that 32% of all patients in the registry are overtriaged and have an ISS of ≤15 (unpublished data). Resuscitation rooms that only have a CT scanner have an inherent risk of unnecessary/needless radiation exposure in overtriaged patients. Depending on the protocol used, estimated effective radiation doses between 10 and 20 mSv have been reported for WB-MSCT scans [5,14-16]. Especially in children and in unrecognized pregnancies, unnecessary radiation exposure due to computed tomography may have deleterious and/or fatal consequences. It is estimated that two thirds of all imaging-induced radiation in the US results from CT scans, with a mortality rate of 12.5 deaths per 10,000 for each CT scan [17]. In contrast, the radiation exposure of conventional radiographs of the thorax (0.07 mSv) and pelvis (0.86 mSv) is much lower [15]. Following the protocol of conventional radiographs used prior to WB-MSCT, a quick decision determining that either the patient is stable enough for further WB-MSCT diagnosis or treatment has to be interrupted so that emergency operations can be performed according to the clinical examination, blood gas analysis, vital parameters, FAST scan, and conventional radiographs of the thorax and pelvis. All of the patients included to this study received these additional diagnostic procedures (clinical examination, vital parameters, FAST scan, and blood gas analysis). Intraabdominal bleeding, hemothorax or pneumothorax, as well as open pelvic ring fractures can be found. Relevant information regarding the need for the early insertion of a chest tube or a pelvic clamp is readily available. If the patient is stable enough for a further WB-MSCT diagnosis, a WB-MSCT scan can be performed. Karlo et al. evaluated different methods of WB-MSCT scans in polytrauma patients and found optimal scan times ranging from 354 to 400 s, depending on the scan protocol that was used [14]. Other authors report CT scan times from 300 to 1,200 s [15,18].

### Treatment time

The time needed in the resuscitation room was significantly shorter in group II, but the time for further treatment, including arrival to the ICU or OR, was the same in both groups. Our data show that the time for the primary and secondary survey was 8 min longer in patients from group I. The delay in this group can be explained by additional transport time to the CT suite and the time required to transfer and set up the patient on the CT table. Considering transportation time from the site of the accident to the hospital and in-hospital transportation time (to the OR or ICU), the additional 8 min only play a minor role in the overall time before further treatment in the ICU or OR. Another important fact is that patients in group I are considered to be stable enough for transportation to the CT suite. The management of a stable patient might be longer compared to a life-threatening situation in an unstable patient. This explains equal arrival times in the OR or ICU, despite a slightly longer examination time in the resuscitation room in group II. The decision to perform a WB-MSCT might also be easier for unstable patients when a CT scanner is available in the resuscitation room. This observation may have led to the faster overall resuscitation room times in group II. If the resuscitation room treatment was interrupted for emergency operations, patients in group II had conventional radiographs available in the OR. The fact that the time to the OR and the ICU did not differ in both groups shows that transportation to the OR and the ICU takes much longer in most hospitals; therefore, a few minutes won in the resuscitation room does not significantly influence the overall treatment time. An argument for the initial use of WB-MSCT is that the patient can be assessed and treated faster with more knowledge regarding the injury. Our data do not support this algorithm concerning treatment time and mortality.

### Availability of WB-MSCT

Many trauma centers with severely injured patients do not have a WB-MSCT scanner available in the resuscitation room. In Germany, only 21% of all supraregional and 5% of all regional trauma centers have a CT scanner located in the resuscitation room. Conventional radiographs are available in the resuscitation room in 83% of all major and in 57.7% of all minor trauma centers. Harris et al. compared the outcomes of patients with traumatic head and brain injuries between hospitals in developed and developing countries. Hospitals in developed countries have more resources available for the treatment of trauma patients (CT, ICU). The use of cranial CT as well as the number of patients admitted to the ICU was significantly higher in developed countries, but the overall mortality rate did not differ significantly [19]. An algorithm that combines conventional radiographs and WB-MSCT is available in every major trauma center with the same outcome compared to the single use of WB-MSCT as shown in this study.

### Limitations

In our study, we compared patients who received an initial conventional radiograph followed by a WB-MSCT to patients who received an initial WB-MSCT. Some limitations might bias the results. The study was not done prospectively and the participating hospitals choose their own diagnostic workup, which is not standardized. We do not have information about structural differences of the different trauma centers, for example, the location of a CT scanner in the resuscitation room or the mean transportation time from the resuscitation room to a CT scanner. In addition, we do not have information about different CT protocols used by the participating hospitals. Potentially different intercenter consistency in grading injuries (abbreviated injury scale or injury severity score) might also bias the results, as described in other studies [1]. This setting excludes hospitals that do not use a WB-MSCT diagnosis in polytrauma management. The comparison of patients who received a WB-MSCT to patients with selective CT scans or conventional radiographs is difficult. More severe injuries, such as lung contusion or liver laceration, were found in the WB-MSCT group and might have been overlooked if a CT scan of this area was not performed.

## Conclusions

Emergency room protocols that use initial conventional radiographs prior to WB-MSCT have comparable results regarding mortality compared to protocols that use WB-MSCT as the initial diagnostic tool. Furthermore, the emergency room team can perform life-savings procedures like chest-tube insertion, thoracotomy, and cardiopulmonary resuscitation immediately. Especially in patients *in extremis*, surgical procedures and diagnostic work-up can be performed simultaneous without wasting precious time. Nevertheless, randomized multi-center trials are needed to determine the comparability and effectiveness of these algorithms.
